# Prognostic significance of T‐cell–inflamed gene expression profile and PD‐L1 expression in patients with esophageal cancer

**DOI:** 10.1002/cam4.4333

**Published:** 2021-10-24

**Authors:** Torben Steiniche, Sun Young Rha, Hyun Cheol Chung, Jeanette Baehr Georgsen, Morten Ladekarl, Marianne Nordsmark, Marie Louise Jespersen, Hyo Song Kim, Hyunki Kim, Carly Fein, Laura H. Tang, Ting Wu, Matthew J. Marton, Senaka Peter, David P. Kelsen, Geoffrey Ku

**Affiliations:** ^1^ Department of Pathology Aarhus University Hospital Aarhus Denmark; ^2^ Division of Medical Oncology, Yonsei Cancer Center Yonsei University College of Medicine Seoul South Korea; ^3^ Department of Oncology, Clinical Cancer Research Center Aalborg University Hospital Aalborg Denmark; ^4^ Department of Oncology Aarhus University Hospital Aarhus Denmark; ^5^ Department of Pathology, Yonsei Cancer Center Yonsei University College of Medicine Seoul South Korea; ^6^ Gastrointestinal Oncology Service, Department of Medicine Memorial Sloan Kettering Cancer Center New York New York USA; ^7^ Department of Pathology Memorial Sloan Kettering Cancer Center New York New York USA; ^8^ BARDS‐Epidemiology Merck & Co., Inc. Kenilworth New Jersey USA; ^9^ Department of Translational Medicine Merck & Co., Inc. Kenilworth New Jersey USA

**Keywords:** adenocarcinoma, retrospective study, squamous cell carcinoma, T‐lymphocytes

## Abstract

**Purpose:**

The ability of the T‐cell–inflamed gene expression profile (GEP) to predict clinical outcome in esophageal cancer (EC) is unknown. This retrospective observational study assessed the prognostic value of GEP and programmed death ligand 1 (PD‐L1) expression in patients with EC treated in routine clinical practice.

**Methods:**

Tumor samples of 294 patients from three centers in Denmark, South Korea, and the United States, collected between 2005 and 2017, were included. T‐cell–inflamed GEP score was defined as non‐low or low using a cutoff of −1.54. A combined positive score (CPS) ≥10 was defined as PD‐L1 expression positivity. Associations between overall survival (OS) and GEP status and PD‐L1 expression were explored by Cox proportional hazards models adjusting for age, sex, histology, stage, and performance status.

**Results:**

Median age was 65 years; 63% of patients had adenocarcinoma (AC) and 37% had squamous cell carcinoma (SCC). Thirty‐six percent of tumors were GEP non‐low, with higher prevalence in AC (46%) than SCC (18%). Twenty‐one percent were PD‐L1–positive: 32% in South Korean samples versus 16% in non‐Asian samples and 26% in SCC versus 18% in AC. GEP scores and PD‐L1 CPS were weakly correlated (Spearman’s *R* = 0.363). OS was not significantly associated with GEP status (non‐low vs low; adjusted hazard ratio, 0.91 [95% CI, 0.69–1.19]) or PD‐L1 expression status.

**Conclusion:**

Neither GEP nor PD‐L1 expression was a prognostic marker in Asian and non‐Asian patients with EC.

## Graphical abstract

In a cohort of 294 patients with esophageal adenocarcinoma and esophageal squamous cell carcinoma from Denmark, South Korea, and the United States, T‐cell–inflamed gene expression profile (GEP) score (non‐low vs low) and PD‐L1 expression status (CPS ≥10 vs CPS <10) showed no significant association with overall survival. Further research investigating prognostic biomarkers in esophageal cancer to inform patient selection for immunotherapy treatment and improve clinical outcomes remains warranted.

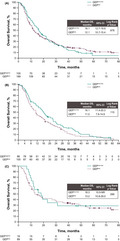

## 1 INTRODUCTION

Esophageal cancer (EC) is the sixth leading cause of cancer‐related death and the seventh most common cancer worldwide.^1^ Risk factors for the two main histologic subtypes of EC, squamous cell carcinoma (SCC) and adenocarcinoma (AC), are different. For example, SCC is associated with tobacco use, alcohol consumption (and mutations in enzymes that metabolize alcohol), low socioeconomic status, poor oral hygiene, and nutritional deficiencies, whereas AC is associated with gastroesophageal reflux disease (GERD), Barrett's esophagus, obesity, tobacco use, and a diet low in vegetables and fruit.^1^, ^2^ SCC represents the majority of EC cases globally and is predominant from the Middle East to central and eastern Asia. The incidence of AC, however, has been increasing in Western countries, primarily in association with increasing rates of obesity and GERD.^3^ Regardless of subtype and region, EC is generally diagnosed at a late stage and patients have a poor prognosis.^3^

A major characteristic of EC is tumor‐induced chronic inflammation, and the presence of tumor‐infiltrating lymphocytes has been associated with improved prognosis.^4^, ^5^ Recently, immune checkpoint inhibition through antibodies that block programmed death 1 (PD‐1) or programmed death ligand 1 (PD‐L1)—signaling proteins that suppress T‐cell migration and proliferation—have led to improvements in patient survival in EC and various other cancers.^6^, ^7^, ^8^, ^9^, ^10^

A recent study^11^ identified a pan‐tumor T‐cell–inflamed gene expression profile (GEP) of 18 genes that predicted clinical response to PD‐1–directed immune checkpoint blockade. This GEP includes interferon‐γ–responsive genes associated with antigen presentation, chemokine expression, cytotoxic activity, and adaptive immune resistance. In a retrospective analysis of tumor samples from anti–PD‐1–treated patients, T‐cell–inflamed GEP was associated with clinical outcomes following pembrolizumab treatment in a pan‐tumor cohort that included EC and in single‐indication cohorts with head and neck squamous cell carcinoma and melanoma.^12^

Higher expression of PD‐L1 might be a prognostic marker and might provide a rationale for inhibiting PD‐L1 in EC. A recent meta‐analysis of 19 studies (*N* = 3306) showed that PD‐L1 overexpression, defined by each study’s cut‐off values, was seen in 1052 patients overall (31.8%; range in individual studies, 14.5%–63.3%) and may be associated with worse survival in EC.^13^ However, to date, most studies on PD‐L1 expression and prognosis have been performed in Asian populations, predominantly with the SCC subtype, and have also used various methodologies for assessing PD‐L1 status.^13^ As such, the prognostic impact of PD‐L1 expression in Western and AC populations is not well‐understood.

High PD‐L1 expression has been related to a greater treatment response to the anti–PD‐1 agent pembrolizumab in patients with refractory advanced EC.^8^, ^14^ Furthermore, an analysis of PD‐L1 expression and T‐cell–inflamed GEP in tumor samples from pembrolizumab‐treated patients with 20 different types of locally advanced or metastatic solid cancers, including EC, has demonstrated greater response in tumors with higher PD‐L1 expression and with higher T‐cell–inflamed GEP.^15^ The prognostic value of T‐cell–inflamed GEP and PD‐L1 expression in patients with EC treated in routine clinical practice, however, is unclear.

The objectives of this study were to characterize T‐cell–inflamed GEP and PD‐L1 expression in patients with locally advanced unresectable or metastatic EC by clinicopathologic characteristics, including of the AC or SCC histologic subtype, and geographic location and to evaluate their prognostic significance in EC.

## 2 METHODS

### 2.1 Study design

This was a retrospective, observational study to examine archived tumor tissue samples from patients with locally advanced unresectable or metastatic EC. Tumor tissue samples collected between 2005 and 2017 were procured from Aarhus University Hospital (Denmark), Yonsei Cancer Center (South Korea), and Memorial Sloan Kettering Cancer Center (United States). Samples were obtained from both AC (esophageal and Siewert type 1 esophagogastric junction [EGJ]) and SCC (esophageal) tumors. Patient clinicopathologic data were obtained by review of medical records. A waiver of patient consent was requested from the ethics review committee or the institutional review board at each institution given that this was a retrospective study with no active recruitment of patients.

### 2.2 Key eligibility criteria

Key inclusion criteria included age ≥18 years at diagnosis, histologically or cytologically confirmed locally advanced unresectable or metastatic AC or SCC of the esophagus or Siewert type I AC of the EGJ, availability of formalin‐fixed paraffin‐embedded (FFPE) archival tissue sample for analysis, and detailed treatment and follow‐up information including OS data. Patients who had received an anti–PD‐1/PD‐L1 or anti–CTLA‐4 agent were excluded.

### 2.3 Assessments

#### 2.3.1 T‐cell–inflamed GEP

The T‐cell–inflamed GEP test used in this study is based on the previously described research use assay, which provides a measure of tumor inflammation across multiple solid tumor types,^15^, ^16^ but was recalibrated to accommodate use as an investigational in vitro diagnostic test. The T‐cell–inflamed GEP score was derived from an 18‐gene signature measured using extracted tumor RNA from FFPE slides. Each FFPE block was reviewed and verified to meet the tissue requirements for the test. A hematoxylin and eosin (H&E) slide, adjacent to the series of unstained slides, was assessed by a pathologist to have a minimum tumor surface area of 2 mm^2^ and ≥10% tumor cellularity within the tumor area. The total number of 5 µm slides used per sample was determined by targeting a total surface area (area per slide x number of slides) of 24 mm^2^. The H&E slide was assumed to be representative of the block and, thus, the unstained slides were not assessed separately.

RNA integrity was assessed using two quality controls: concentration using A260 ≥5 ng/µl and purity of an A260/A280 ratio between 1.7–2.3. Because ribosomal RNA degrades during formalin fixation and paraffin embedding, the RNA integrity number (RIN) is typically not a useful measure of RNA integrity for FFPE tissue and was thus not measured in this analysis.

The extracted RNA was analyzed on the NanoString Counter platform (NanoString Technologies, Seattle, WA, USA)^16^, ^17^, ^18^ and a customized gene expression panel manufactured under Good Manufacturing Practice. The customized panel of genes represents the tumor inflammation signature, which provides a measure of tumor inflammation across multiple solid tumor types.^15^, ^16^ GEP score was calculated as a weighted sum of normalized expression values of 18 genes (*CCL5*, *CD27*, *CD274 [PD*‐*L1]*, *CD276 [B7*‐*H3]*, *CD8A*, *CMKLR1*, *CXCL9*, *CXCR6*, *HLA*‐*DQA1*, *HLA*‐*DRB1*, *HLA*‐*E*, *IDO1*, *LAG3*, *NKG7*, *PDCD1LG2 [PD*‐*L2]*, *PSMB10*, *STAT1*, and *TIGIT*).^15^, ^16^ GEP status was defined using a cut‐off score of −1.54, such that GEP^low^ was defined as <−1.54 and GEP^non−low^ was defined as ≥−1.54.

#### 2.3.2 PD‐L1 expression

Tumor samples were analyzed for PD‐L1 expression by immunohistochemistry (IHC) staining using the PD‐L1 IHC 22C3 pharmDx (Agilent Technologies, Carpinteria, CA, USA). PD‐L1 expression was reported as combined positive score (CPS), defined as the number of PD‐L1–positive cells (tumor cells, lymphocytes, and macrophages) divided by the total number of tumor cells, multiplied by 100. PD‐L1–positive expression was defined as CPS ≥10, consistent with the definition in pembrolizumab EC clinical trials.^14^, ^19^

### 2.4 Endpoint

The primary endpoint was OS, calculated from the date of diagnosis or the start date of first‐line therapy to the date of death from any cause; data were censored if death was not documented at the time of last follow‐up.

### 2.5 Statistical analysis

Associations between GEP status and PD‐L1 expression with clinicopathologic characteristics were explored as categorical variables using chi‐square tests and multiple logistic regression models. The relationship between OS and GEP status or PD‐L1 expression was analyzed by Kaplan–Meier methods using the log‐rank test. Multivariate Cox proportional hazards models were also used with adjustments for age, gender, histology, stage, and Eastern Cooperative Oncology Group performance status (ECOG PS). Covariates included in the final models were based on a stepwise variable selection process. The association between GEP score and PD‐L1 expression was measured using Spearman's rank correlation coefficients.

## 3 RESULTS

In total, 294 patients with tumor tissue samples of AC and SCC histology subtypes and available GEP score (Table 1) and PD‐L1 CPS data (Table 2) were included in the analysis. The median age of these patients was 65 years (range, 38–88); most were male (85%) and 7%, 9%, 21%, and 58% had stage I, II, III and IV disease, respectively (American Joint Committee on Cancer TNM stage information could not be assessed in 5%). There were 120 patients (41%) who were current or ex‐smokers and 57 (19%) who never smoked; smoking status was not available or unknown for 117 patients (40%). The proportions of patients with AC and SCC histologic subtypes were 63% and 37%, respectively. Consistent with the global burden of esophageal cancer by subtype, AC was most common (169/210; 80%) among patients from non‐Asian countries (Denmark and United States), and SCC most common (67/84; 80%) among patients from South Korea.

**TABLE 1:** Association of GEP status with clinicopathologic characteristics

Characteristics	Total patients	GEP expression		Chi‐square test *p* value*
		GEP^non−low^	GEP^low^	
Overall, *n* (%)	294 (100)	105 (35.7)	189 (64.3)	NA
Age (years)				
Median (range)	65 (38–88)			
<65	136 (46.3)	48 (35.3)	88 (64.7)	0.889
≥65	158 (53.7)	57 (36.1)	101 (63.9)	
Gender, *n* (%)				
Male	249 (84.7)	84 (33.7)	165 (66.3)	0.096
Female	45 (15.3)	21 (46.7)	24 (53.3)	
Smoking history^a^, *n* (%)				
Never	57 (19.4)	22 (38.6)	35 (61.4)	0.888
Ex‐smoker/current	120 (40.8)	45 (37.5)	75 (62.5)	
Tumor site^b^, *n* (%)				
Esophagus	225 (76.5)	74 (32.9)	151 (67.1)	0.173
EGJ	33 (11.2)	14 (42.4)	19 (57.6)	
Other^c^	36 (12.2)	17 (47.2)	19 (52.8)	
ECOG PS^d^, *n* (%)				
0	117 (39.8)	43 (36.8)	74 (63.2)	0.811
≥1	150 (51.0)	53 (35.3)	97 (64.7)	
Region, *n* (%)				
Denmark	123 (41.8)	45 (36.6)	78 (63.4)	0.114
South Korea	84 (28.6)	23 (27.4)	61 (72.6)	
United States	87 (29.6)	37 (42.5)	50 (57.5)	
Asian (South Korea)	84 (28.6)	23 (27.4)	61 (72.6)	0.059
Non‐Asian (Denmark and United States)	210 (71.4)	82 (39.0)	128 (61.0)	
Histology, *n* (%)				
Adenocarcinoma	186 (63.3)	86 (46.2)	100 (53.8)	<0.001
Squamous cell carcinoma	108 (36.7)	19 (17.6)	89 (82.4)	
Histologic grade^e^, *n* (%)				
Well/moderately differentiated	144 (49.0)	48 (33.3)	96 (66.7)	0.035
Poorly differentiated/undifferentiated	81 (27.6)	39 (48.1)	42 (51.9)	
Signet ring cell	6 (2.0)	4 (66.7)	2 (33.3)	
Clinical stage^f^, *n* (%)				
I (I, IB)	20 (6.8)	4 (20.0)	16 (80.0)	0.161
II (II, IIA, IIB, I‐II)	25 (8.5)	8 (32.0)	17 (68.0)	
III (III, IIIA, IIIA‐IIIB, IIIB, IIIC, II‐III)	63 (21.4)	29 (46.0)	34 (54.0)	
IV	170 (57.8)	60 (35.3)	110 (64.7)	

Abbreviations: ECOG PS, Eastern Cooperative Oncology Group performance status; EGJ, esophagogastric junction; GEP, T‐cell–inflamed gene expression profile; NA, not applicable.

^a^ Data on smoking history were not available for all Korean patients (*n* = 84) and unknown for 33 patients.

^b^ Location of the biopsy specimen, not necessarily that of the primary tumor.

^c^ Metastatic site (solid organ) or lymph node in particular. These are excluded from the chi‐square test.

^d^ Twenty‐seven patients had unknown or missing ECOG PS.

^e^ Sixty‐three patients had unknown histologic grade.

^f^ Sixteen patients had unknown or missing clinical stage.

* *p* value to test for difference between the subgroups in the overall cohort.

**TABLE 2:** Association of PD‐L1 expression with clinicopathologic characteristics

Characteristics	Total patients	PD‐L1 expression		Chi‐square test *p* value*
		PD‐L1 CPS ≥10	PD‐L1 CPS <10	
Overall, *n* (%)	294	61 (20.7)	233 (79.3)	NA
Age (years)				
Median (range)	65 (38–88)			
<65	136 (46.3)	22 (16.2)	114 (83.8)	0.073
≥65	158 (53.7)	39 (24.7)	119 (75.3)	
Gender, *n* (%)				
Male	249 (84.7)	52 (20.9)	197 (79.1)	0.893
Female	45 (15.3)	9 (20.0)	36 (80.0)	
Smoking history^a^, *n* (%)				
Never	57 (19.4)	10 (17.5)	47 (82.5)	0.665
Ex‐smoker/current	120 (40.8)	18 (15.0)	102 (85.0)	
Tumor site^b^, *n* (%)				
Esophagus	225 (76.5)	49 (21.8)	176 (78.2)	0.112
EGJ	33 (11.2)	9 (27.3)	24 (72.7)	
Other^c^	36 (12.2)	3 (8.3)	33 (91.7)	
ECOG PS^d^, *n* (%)				
0	117 (39.8)	30 (25.6)	87 (74.4)	0.131
≥1	150 (51.0)	27 (18.0)	123 (82.0)	
Region, *n* (%)				
Denmark	123 (41.8)	23 (18.7)	100 (81.3)	0.005
South Korea	84 (28.6)	27 (32.1)	57 (67.9)	
United States	87 (29.6)	11 (12.6)	76 (87.4)	
Asian (South Korea)	84 (28.6)	27 (32.1)	57 (67.9)	0.002
Non‐Asian (Denmark and United States)	210 (71.4)	34 (16.2)	176 (83.8)	
Histology, *n* (%)				
Adenocarcinoma	186 (63.3)	33 (17.7)	153 (82.3)	0.095
Squamous cell carcinoma	108 (36.7)	28 (25.9)	80 (74.1)	
Histologic grade^e^, *n* (%)				
Well/moderately differentiated	144 (49.0)	32 (22.2)	112 (77.8)	0.427
Poorly differentiated/undifferentiated	81 (27.6)	18 (22.2)	63 (77.8)	
Signet ring cell	6 (2.0)	—	6 (100.0)	
Clinical stage^f^, *n* (%)				
I (I, IB)	20 (6.8)	3 (15.0)	17 (85.0)	0.757
II (II, IIA, IIB, I‐II)	25 (8.5)	5 (20.0)	20 (80.0)	
III (III, IIIA, IIIA‐IIIB, IIIB, IIIC, II‐III)	63 (21.4)	15 (23.8)	48 (76.2)	
IV	170 (57.8)	31 (18.2)	139 (81.8)	

Abbreviations; CPS, combined positive score; ECOG PS, Eastern Cooperative Oncology Group performance status; EGJ, esophagogastric junction; NA, not applicable; PD‐L1, programmed death ligand 1.

^a^ Data on smoking history were not available for all Korean patients (*n* = 84) and unknown for 33 patients.

^b^ Location of the biopsy specimen, not necessarily that of the primary tumor.

^c^ Metastatic site (solid organ) or lymph node in particular.

^d^ Twenty‐seven patients had unknown or missing ECOG PS.

^e^ Sixty‐three patients had unknown histologic grade.

^f^ Sixteen patients had unknown or missing clinical stage.

* *p* value to test for difference between the subgroups in the overall cohort.

Tissue for biomarker testing was predominantly biopsy samples (96%); 239 of 294 samples (81%) were collected prior to first‐line therapy initiation. Two hundred seventy‐three patients (93%) were reported to have received first‐line therapy, and 21 patients (7%) did not have a record of receiving antineoplastic treatment. Of the 273 patients who received therapy, 107 (39%) and 49 (18%) received second‐line and third‐line therapy, respectively. As first‐line therapy, 189 patients (69%) received chemotherapy (in combination with trastuzumab in human epidermal growth factor receptor‐2 [HER2]–positive AC), 80 (29%) received chemoradiation, and 4 (1.5%) received radiation only. Lines of therapy are reported separately for patients with AC and SCC in Table S1.

### 3.1 Association of GEP status and PD‐L1 expression with clinicopathologic characteristics

Overall, 36% of patients had a GEP status of non‐low. Associations between GEP status and clinicopathologic characteristics in the overall study population are summarized in Table 1. GEP status was significantly associated with histology (*p* < 0.001) and histologic grade (*p* = 0.035), with GEP^non−low^ status more common in patients with the AC versus the SCC subtype and in poorly differentiated/undifferentiated versus well‐differentiated tumors. In a multiple regression analysis that included age, gender, geographic location, ECOG PS, histologic grade, histology, and stage, histology (AC vs. SCC) remained statistically significant for association with GEP status (adjusted odds ratio [OR], 0.248; 95% CI, 0.139–0.441; *p* < 0.001). A plot of GEP score distribution by histologic subtype can be found in Figure S1. There were no statistically significant associations between GEP status and age, gender, smoking history, or clinical stage (Table 1).

Based on a cutoff of CPS ≥10, 21% of patients had PD‐L1–positive tumors (Table 2). There were no statistically significant associations between PD‐L1 expression and age, gender, smoking history, tumor site, or grade and stage. Higher PD‐L1 expression was observed in tumor samples of patients from South Korea (32%) compared with those from non‐Asian countries (Denmark, 19%; United States, 13%) (*p* = 0.005; Table 2). Although not statistically significant, PD‐L1 overexpression was more frequent in SCC (26%) than in AC (18%) samples, which may account for the difference in geographic region observed between the South Korean patients (80% had SCC tumors) and the non‐Asian patients (80% had AC tumors) (Figure S2). To examine further, we looked at the regional difference in frequency of PD‐L1 expression by histologic subtype. PD‐L1 expression in SCC tumors was significantly more frequent in South Korean than in non‐Asian patients (32.8% vs. 14.6%, respectively; *p* = 0.036) (Table S2), and the proportion of AC tumors that were PD‐L1–positive was numerically greater in South Korean than in non‐Asian patients (29.4% vs. 16.6%, respectively; *p* = 0.186), although the smaller sample size of Asian patients with AC tumors should be noted (Table S3). A statistically significant association between PD‐L1 expression and HER2 status was seen in the subset of patients with AC tumors. HER2+ tumors were less likely to be PD‐L1 positive than HER2– tumors (8.7% vs. 22.7%). When using a different CPS cutoff (CPS ≥1) to define PD‐L1 positivity, patients’ SCC and AC tumors had comparable frequencies of PD‐L1 positivity overall (61% vs. 64%, respectively). However, PD‐L1 positivity was higher in SCC and AC tumor samples from South Korean patients than in those from non‐Asian patients, indicating that the higher CPS cutoff does not explain the association between PD‐L1 expression and geographic location.

### 3.2 Relationship between GEP score and PD‐L1 CPS

**FIGURE 1:**
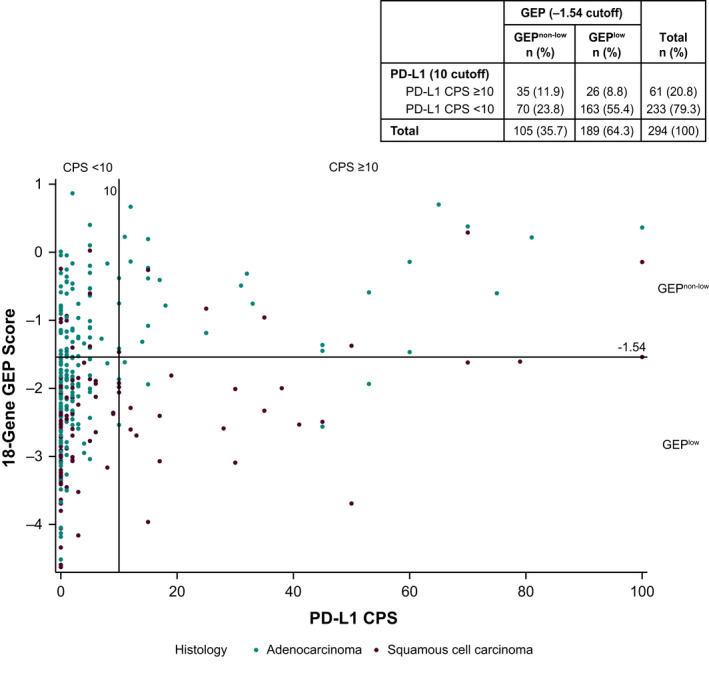
Scatterplot of GEP score versus PD‐L1 CPS in patients with adenocarcinoma and squamous cell carcinoma and distribution of GEP according to PD‐L1 expression level. CPS, combined positive score; GEP, T‐cell–inflamed gene expression profile; PD‐L1, programmed death ligand 1

Correlation analysis demonstrated a mild correlation between GEP score and PD‐L1 CPS (Spearman's Rho = 0.363; *p* < 0.0001). With a cut‐off of −1.54, expression of GEP was predominantly low among the total population (189/294; 64%): 55% of patients had GEP^low^ and PD‐L1 CPS <10, and 8.8% of patients had GEP^low^ and PD‐L1 CPS ≥10; 24% of patients had GEP^non−low^ and CPS <10, and 12% of patients had GEP^non−low^ and CPS ≥10 (*p* < 0.001) (Figure 1).

### 3.3 Association between tumor site and overall survival

In this cohort, there was no statistically significant difference in OS between the samples from patients with EC and those with cancer of the EGJ (*p* = 0.3343).

### 3.4 Association of GEP status and PD‐L1 expression with overall survival

**FIGURE 2:**
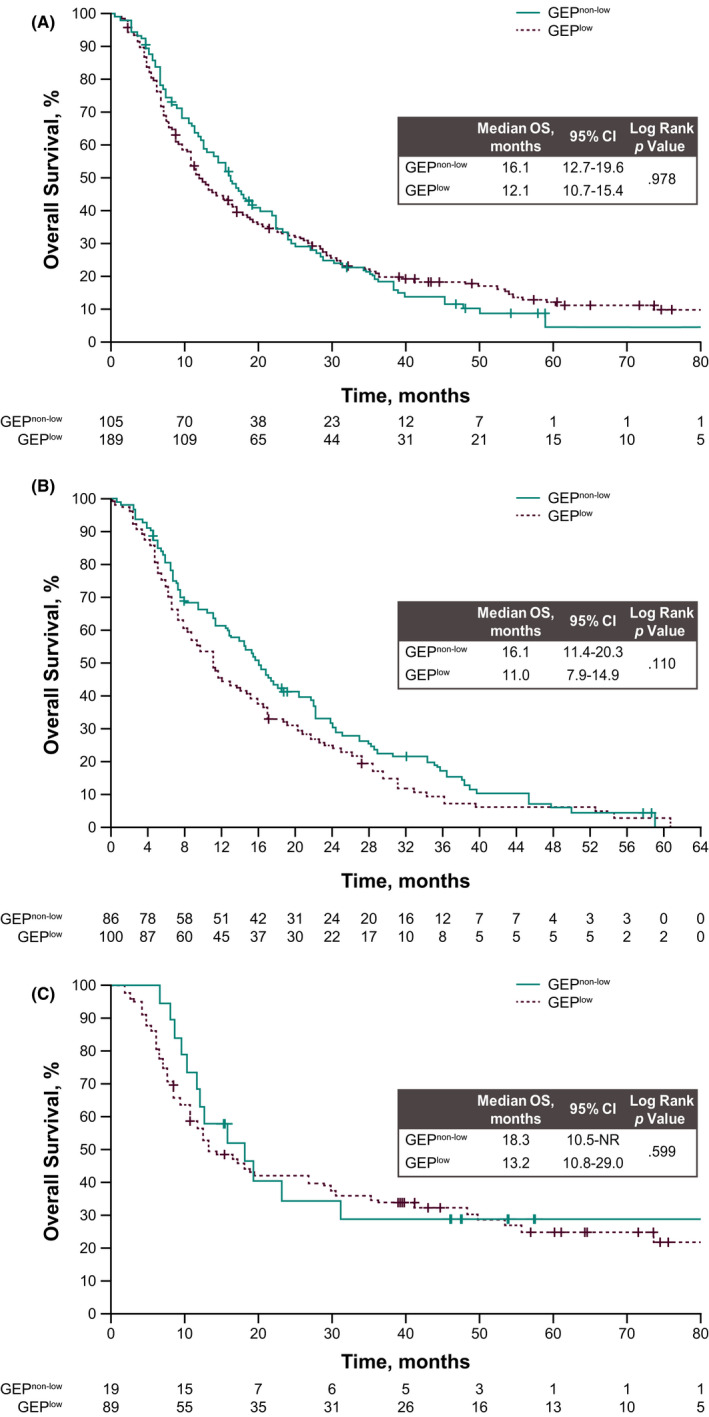
Kaplan–Meier estimates for overall survival (A) from date of diagnosis by GEP status (non‐low vs. low) overall and in patients with (B) adenocarcinoma and (C) squamous cell carcinoma. GEP, T‐cell–inflamed gene expression profile

Median OS from diagnosis in all patients (*N* = 294) was 13.7 months (95% CI, 12–16 months). There was no significant difference in OS between patients with GEP^non−low^ (16.1 months) and GEP^low^ (12.1 months) (log‐rank *p* = 0.98) (Figure 2A). In a Cox proportional hazards model, the adjusted hazard ratio [aHR] for GEP^non−low^ versus GEP^low^ was 0.91 (95% CI, 0.69–1.19) adjusted for stage, ECOG PS, and PD‐L1 expression status. Similarly, subgroup analyses did not demonstrate any statistically significant association between GEP status and OS in either histologic subtype (AC: aHR 0.89; 95% CI, 0.65–1.23; SCC: aHR, 0.78; 95% CI, 0.43–1.44) (Figure 2B, C). Results were similar when calculated from the date of first‐line treatment (*n* = 273; Figure S3). Median OS from the date of first‐line treatment was 10.7 months (95% CI, 9–13 months).

Although no association was observed between GEP status and OS among patients with early‐stage disease (stages I‐III; *n* = 108; log rank *p* = 0.083), a trend toward longer survival was observed among patients with advanced‐stage GEP^non−low^ (stage IV; *n* = 170; log rank *p* = 0.059), but neither difference was significant (Figures S4A,B). No association was observed between GEP status and OS among patients with early‐stage disease (stages I‐III; *n* = 59; log rank *p* = 0.926) or with late‐stage or advanced AC (stage IV; *n* = 125; log rank *p* = 0.193) or among patients with early‐stage SCC (stages I‐III; *n* = 49; log rank *p* = 0.621) or with late‐stage or advanced SCC (IV; *n* = 45; log rank *p* = 0.307; Figures S4C–F).

**FIGURE 3:**
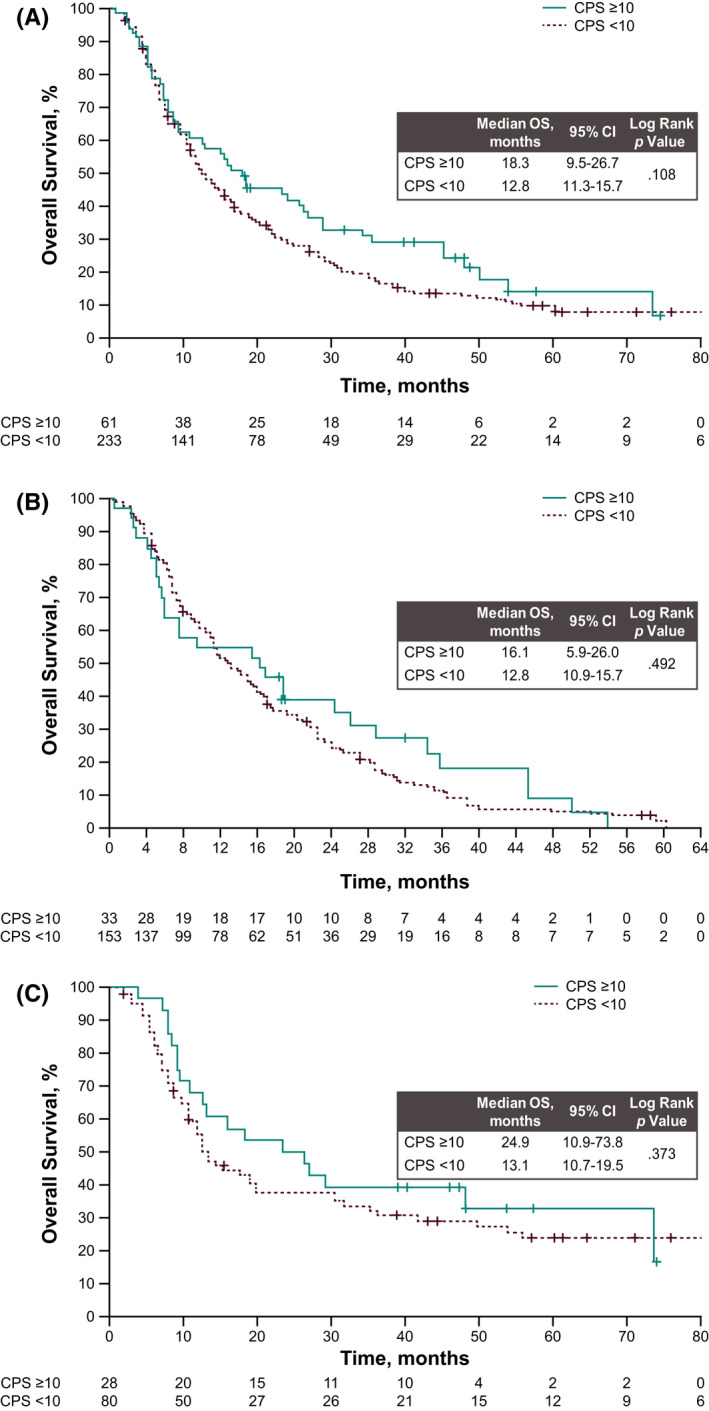
Kaplan–Meier estimates for overall survival (A) from date of diagnosis by PD‐L1 expression (PD‐L1 CPS ≥10 vs. <10) overall and in patients with (B) adenocarcinoma and (C) squamous cell carcinoma. CPS, combined positive score; PD‐L1, programmed death ligand 1

There was also no statistically significant association between OS and PD‐L1 expression (CPS <10 vs. CPS ≥10; aHR, 0.86; 95% CI, 0.62–1.18), adjusted for TNM stage and ECOG PS (Figure 3A). Similar results were observed in subgroup analyses by histologic subtype (AC: aHR 0.86; 95% CI, 0.58–1.29; SCC: aHR, 1.16; 95% CI, 0.66–2.04) (Figure  3B, C).

When analyzing data for patients by GEP and CPS status together, median OS was highest in patients with CPS ≥10 and GEP^non−low^ tumors (18.3 months). Median OS was 17.7 months in patients with CPS ≥10 and GEP^low^ tumors, 15.7 months in patients with CPS <10 and GEP^non−low^ tumors, and 11.7 months in patients with CPS <10 and GEP^low^ tumors (Figure S5).

## 4 DISCUSSION

In this study, we examined T‐cell–inflamed GEP and PD‐L1 expression in a large cohort of patients (*N* = 294) with EC from Denmark, South Korea, and the United States and assessed the association of these factors with OS. More than one‐third of the patients had GEP^non−low^ tumors, and a significantly higher prevalence was found in patients with AC than with SCC (46% vs. 18%). In addition, approximately one‐fifth of the patients had PD‐L1–positive status (CPS ≥10); a higher proportion of patients with PD‐L1–positive expression was observed among the South Korean patients than the non‐Asian population, possibly because a high proportion of South Korean patients had the SCC subtype (80%), which was more likely than the AC subtype to have PD‐L1 positivity (26% vs. 18% for SCC and AC, respectively).

The prevalence of PD‐L1–positive expression in our study (21%) was lower than that reported among patients with EC in the KEYNOTE‐180 and KEYNOTE‐181 clinical trials (48% and 35%, respectively) using the same PD‐L1 IHC 22C3 pharmDx (Agilent Technologies) and cut‐off for assessing PD‐L1 expression.^8^, ^14^, ^20^ This might have been due to the lower proportion of patients with SCC in the current study (37%) compared with KEYNOTE‐180 (52%) and KEYNOTE‐181 (64%).^8^, ^14^ A retrospective analysis of 3342 gastroesophageal tumors found that esophageal SCC tumors had a higher expression of PD‐L1 than AC tumors,^21^ results that are consistent with our findings despite use of a different PD‐L1 IHC antibody (SP142) and a different criterion for overexpression (>5% of cells) and lack of important clinical data‐like stage available for the cohort.

In addition, the number of previous lines of therapy may play a role in the prevalence of PD‐L1 positivity. Lower prevalence of PD‐L1 positivity and other lymphocyte markers has been observed in later lines of therapy in breast cancer.^22^ However, in preclinical and clinical studies of EC, SCC, ovarian cancer, and non–small cell lung carcinoma,^23^, ^24^, ^25^, ^26^, ^27^ higher prevalence of PD‐L1 positivity has been seen in later lines of therapy, particularly in patients who previously received platinum therapy. The age of the samples used in this retrospective study may also play a role. Although there was no set age for sample rejection in the study protocol, the PD‐L1 IHC assay kit is recommended for use on tumor blocks <5 years of age because tumor blocks ≥5 years of age may result in less staining or in loss of immunoreactivity. Finally, samples from EC are also smaller and difficult to procure, making immunohistochemistry assays challenging.

A strength of this study is the inclusion of Asian and non‐Asian patients. The inclusion of subgroup analyses also provided insight into the AC and SCC subtypes. Moreover, this study reports real‐world data on the frequencies of PD‐L1 positivity, with samples analyzed centrally at the same laboratory, thus strengthening any findings of differences between patients from different geographic areas. The same assay that was used in the KEYNOTE clinical trials in patients with advanced EC^8^, ^14^ was used in the current study, providing consistency between real‐world and clinical trial data. Our study also demonstrated a mild correlation between GEP score and PD‐L1 CPS score (*r* = 0.36), comparable to that reported in the KEYNOTE‐028 study patients with multiple tumor types treated with pembrolizumab, which found a moderate but significant correlation (*r* = 0.40; *p* < 0.001; *n* = 151).^15^ Overall, there were no significant differences in OS between patients with GEP^non−low^ and GEP^low^ status or between patients with PD‐L1 CPS ≥10 and CPS <10, indicating that these are not markers of OS. Nevertheless, OS was numerically highest in patients with both CPS ≥10 and GEP^non−low^ status.

Notably, the PD‐L1 results also contrasted with those of recent meta‐analyses demonstrating that PD‐L1 expression in tumor cells detected by IHC was associated with worse OS in EC.^13^, ^28^ Both meta‐analyses showed that PD‐L1 was significantly associated with poor prognosis for OS in EC patients; however, nearly all the studies in both meta‐analyses were in Asian patients (17/19 in one meta‐analysis,^13^ 12/13 in the other^28^) and patients with SCC (all in one meta‐analysis,^13^ 10/13 in the other^28^). This illustrates the paucity of data on PD‐L1 expression in AC and in non‐Asian populations, where the prognostic significance of PD‐L1 remains unclear. The results of the present study provide preliminary insight into the possible role of PD‐L1 expression as a prognostic marker in the AC subtype. Further studies would be required to investigate this hypothesis.

In addition, studies included in these meta‐analyses^13^, ^28^ were not standardized with regard to methodology of PD‐L1 expression assessment, including IHC techniques, primary antibody used, antibody concentration used, and cut‐off values for positive expression. PD‐L1 status can also be influenced by variations in tissue processing between studies, interobserver variation, and tumor heterogeneity, all of which could contribute to variability between studies. In the current real‐world study, the methodology was identical to that used prospectively in studies of pembrolizumab in EC and that is required as part of the US Food and Drug Administration approval of pembrolizumab in SCC. These variations may account for differences in clinical outcomes observed between this study and the studies included in the meta‐analyses.^13^, ^28^ To our knowledge, no other study has evaluated an Asian and non‐Asian population using a validated PD‐L1 immunohistochemistry method and central GEP assessment.

However, this study is also subject to the limitations inherent to a retrospective observational design. Although most patients (79%) had stage III or IV disease, a small proportion of samples from patients with stage I and II disease were included, and no information was captured to clarify their resection status. Treatment patterns may be heterogeneous from retrospective chart review, which is limited by data availability and quality. In addition, the exact location of the primary tumor (proximal vs. mid vs. distal esophagus vs. EGJ) was not recorded. While there was no difference in GEP status or PD‐L1 CPS between patients who identified as smokers (former or current) and patients who never smoked, smoking history was missing from Korean patients. As a result, this limits the generalizability of these results to the Asian population or SCC population, of which Korean patients predominated. Furthermore, analysis of association of GEP status and PD‐L1 expression with OS was limited in that Cox proportional hazards modeling did not adjust for treatment.

## 5 CONCLUSION

This observational study showed that GEP status and PD‐L1 expression were not prognostic factors of OS in this cohort of Asian and non‐Asian EC patients with AC and SCC. Additionally, our study was not statistically powered to evaluate the combination of these two biomarkers but this strategy is worthy of further investigation. Our results highlight the continued need to identify novel prognostic biomarkers for patients with EC.

## DISCLOSURES

Torben Steiniche: Research funding and honoraria– Merck Sharp & Dohme Corp., a subsidiary of Merck & Co., Inc., Kenilworth, NJ, USA. Sun Young Rha has nothing to disclose. Hyun Cheol Chung: Research funding – Amgen, BeiGene, Merck Serono, Eli Lilly, Taiho, BMS Ono, GlaxoSmithKline. Consulting/advisory – Amgen, BeiGene, Merck Serono, Eli Lilly, Taiho, Celltrion, Quintiles, Gloria, Bristol Myers Squibb, Merck Sharp & Dohme Corp., a subsidiary of Merck & Co., Inc., Kenilworth, NJ, USA. Honoraria – Merck Serono, Eli Lilly. Jeanette Baehr Georgsen has nothing to disclose. Morten Ladekarl: Honoraria – Celgene, Bayer. Marianne Nordsmark: Stock ownership – Novo Nordisk. Marie Louise Jespersen: Consulting/advisory –Merck Sharp & Dohme Corp., a subsidiary of Merck & Co., Inc., Kenilworth, NJ, USA. Hyo Song Kim has nothing to disclose. Hyunki Kim has nothing to disclose. Carly Fein: Employment – Novant Health. Laura H. Tang has nothing to disclose. Ting Wu: Employment – Merck Sharp & Dohme Corp., a subsidiary of Merck & Co., Inc., Kenilworth, NJ, USA. Matthew Marton: Employment – Merck Sharp & Dohme Corp., a subsidiary of Merck & Co., Inc., Kenilworth, NJ, USA. Stock ownership – Merck & Co., Inc., Kenilworth, NJ, USA. Senaka Peter: Employment – Merck Sharp & Dohme Corp., a subsidiary of Merck & Co., Inc., Kenilworth, NJ, USA. Stock ownership – Merck & Co., Inc., Kenilworth, NJ, USA. David Kelsen has nothing to disclose. Geoffrey Ku: Research funding – Arog Pharma, Aduro Biotech, Merck Sharp & Dohme Corp., a subsidiary of Merck & Co., Inc., Kenilworth, NJ, USA, AstraZeneca, Bristol Myers Squibb, Pieris. Consulting/advisory – Eli Lilly, Merck Sharp & Dohme Corp., a subsidiary of Merck & Co., Inc., Kenilworth, NJ, USA, AstraZeneca, Bristol Myers Squibb, Pieris.

## CONFLICT OF INTEREST

All authors declare that there is no conflict interest.

## Supporting information

Tables S1–S3 and Figures S1–S5

## Data Availability

Merck Sharp & Dohme Corp., a subsidiary of Merck & Co., Inc., Kenilworth, NJ, USA (MSD) is committed to providing qualified scientific researchers access to anonymized data and clinical study reports from the company's clinical trials for the purpose of conducting legitimate scientific research. MSD is also obligated to protect the rights and privacy of trial participants and, as such, has a procedure in place for evaluating and fulfilling requests for sharing company clinical trial data with qualified external scientific researchers. The MSD data sharing website (available at: http://engagezone.msd.com/ds_documentation.php) outlines the process and requirements for submitting a data request. Applications will be promptly assessed for completeness and policy compliance. Feasible requests will be reviewed by a committee of MSD subject matter experts to assess the scientific validity of the request and the qualifications of the requestors. In line with data privacy legislation, submitters of approved requests must enter into a standard data‐sharing agreement with MSD before data access is granted. Data will be made available for request after product approval in the US and EU or after product development is discontinued. There are circumstances that may prevent MSD from sharing requested data, including country or region‐specific regulations. If the request is declined, it will be communicated to the investigator. Access to genetic or exploratory biomarker data requires a detailed, hypothesis‐driven statistical analysis plan that is collaboratively developed by the requestor and MSD subject matter experts; after approval of the statistical analysis plan and execution of a data‐sharing agreement, MSD will either perform the proposed analyses and share the results with the requestor or will construct biomarker covariates and add them to a file with clinical data that is uploaded to an analysis portal so that the requestor can perform the proposed analyses.
