# Supplementary material for: Prognostic significance of T‐cell–inflamed gene expression profile and PD‐L1 expression in patients with esophageal cancer
Source: Cancer Med. 2021 Oct 24;10(23):8365–76. doi: 10.1002/cam4.4333 (PMC8633232; doi:10.1002/cam4.4333)
Supplement: Supplementary file 1 — Tables S1–S3 and Figures S1–S5 [file CAM4-10-8365-s001.pdf]

SUPPLEMENTARY TABLES and FIGURES

Prognostic significance of T-cell–inflamed gene expression profile and PD-L1 expression in patients with esophageal cancer.

Supplementary Table S1. Lines of therapy for patients with squamous cell carcinoma and adenocarcinoma

	**SCC Patients N = 108 n (%)**	**AC Patients N = 186 n (%)**
First-line	95 (88.0)	178 (95.7)
Chemotherapy	42 (44.2)	147 (82.6)
Radiation	1 (1.1)	3 (1.7)
Chemoradiation	52 (54.7)	28 (15.7)
Second-line	61 (64.2)	46 (25.8)
Chemotherapy	32 (52.5)	31 (67.4)
Radiation	13 (21.3)	12 (26.1)
Chemoradiation	16 (26.2)	3 (6.5)
Third-line	24 (39.3)	25 (54.3)
Chemotherapy	11 (45.8)	18 (72.0)
Radiation	3 (12.5)	5 (20.0)
Chemoradiation	10 (41.7)	2 (8.0)

Supplementary Table S2. Association of PD-L1 expression with clinicopathologic characteristics among patients with the squamous cell carcinoma subtype

**Characteristics**	**SCC patients**	**PD-L1 expression**		**Chi-square test *p* value***
		**PD-L1 CPS ≥10**	**PD-L1 CPS <10**	
Overall, n (%)	108 (100)	28 (25.9)	80 (74.1)	NA
Age (years)				
<65	47 (43.5)	13 (27.7)	34 (72.3)	.718
≥65	61 (56.5)	15 (24.6)	46 (75.4)	
Gender, n (%)				
Male	93 (86.1)	25 (26.9)	68 (73.1)	.572
Female	15 (13.9)	3 (20.0)	12 (80.0)	
ECOG PS,[a] n (%)				
0	53 (49.1)	19 (35.8)	34 (64.2)	0.025
≥1	40 (37.0)	6 (15.0)	34 (85.0)	
Region, n (%)				
Asian (South Korea)	67 (62.0)	22 (32.8)	45 (67.2)	0.036
Non-Asian (Denmark and United States)	41 (38.0)	6 (14.6)	35 (85.4)	
Histologic grade,[b] n (%)				
Well/moderately differentiated	56 (52.0)	19 (33.9)	37 (66.1)	0.096
Poorly differentiated/undifferentiated	16 (14.8)	2 (12.5)	14 (87.5)	
Clinical stage,[c] n (%)				
I (I, IB)	16 (14.8)	2 (12.5)	14 (87.5)	0.467
II (II, IIA, IIB, I-II)	12 (11.1)	3 (25.0)	9 (75.0)	
III (III, IIIA, IIIA-IIIB, IIIB, IIIC, II-III)	21 (19.4)	7 (33.3)	14 (66.7)	
IV	45 (41.7)	9 (20.0)	36 (80.0)	

**p* value to test for difference between the subgroups in the overall cohort.

[a]15 patients (total) had unknown or missing ECOG PS.

[b]36 patients had unknown histologic grade.

[c]14 patients had unknown or missing clinical stage.

Abbreviations: CPS: combined positive score; ECOG PS: Eastern Cooperative Oncology Group performance status; NA: not applicable; PD-L1: programmed death ligand 1.

Supplementary Table S3. Association of PD-L1 expression with clinicopathologic characteristics among patients with the adenocarcinoma subtype

Characteristics	AC patients	PD-L1 expression		Chi-square test *p* value*
		PD-L1 CPS ≥10	PD-L1 CPS <10	
Overall, n (%)	186 (100)	33 (17.7)	153 (82.3)	
Age (years)				
<65	89 (47.8)	9 (10.1)	80 (89.9)	.009
≥65	97 (52.2)	24 (24.7)	73 (75.3)	
Gender, n (%)				
Male	156 (83.9)	27 (17.3)	129 (82.7)	.724
Female	30 (16.1)	6 (20.0)	24 (80.0)	
Tumor site,[a] n (%)				
Esophagus	118 (63.4)	21 (17.8)	97 (82.2)	.131
EGJ	33 (17.7)	9 (27.3)	24 (72.7)	
Other[b]	35 (18.8)	3 (8.6)	32 (91.4)	
ECOG PS,[c] n (%)				
0	64 (34.4)	11 (17.2)	53 (82.8)	.755
≥1	110 (59.1)	21 (19.1)	89 (80.9)	
Region, n (%)				
Asian (South Korea)	17 (9.1)	5 (29.4)	12 (70.6)	.186
Non-Asian (Denmark and United States)	169 (90.9)	28 (16.6)	141 (83.4)	
Histologic grade,[d] n (%)				
Well/moderately differentiated	88 (47.3)	13 (14.8)	75 (85.2)	.148
Poorly differentiated/undifferentiated	65 (34.9)	16 (24.6)	49 (75.4)	
Signet ring cell	6 (3.2)	—	6 (100)	
Clinical stage,[e] n (%)				
I (I, IB)	4 (2.2)	1 (25.0)	3 (75.0)	.971
II (II, IIA, IIB, I-II)	13 (7.0)	2 (15.4)	11 (84.6)	
III (III, IIIA, IIIA-IIIB, IIIB, IIIC, II-III)	42 (22.6)	8 (19.0)	34 (81.0)	
IV	125 (67.2)	22 (17.6)	103 (82.4)	
HER2 status,[f,g] n (%)				
HER2+	46 (24.7)	4 (8.7)	42 (91.3)	.043
HER2−	97 (52.2)	22 (22.7)	75 (77.3)	

*p value to test for difference between the subgroups in the overall cohort.

[a]Location of the biopsy specimen, not necessarily that of the primary tumor.

[b]Metastatic site (solid organ) or lymph node in particular.

[c]12 patients had unknown or missing ECOG PS.

[d]27 patients had unknown histologic grade.

[e]2 patients had unknown or missing clinical stage.

[f]43 patients had unknown or missing HER2 status.

[g]HER2 status data were available only for the AC subtype.

Abbreviations: CPS: combined positive score; ECOG PS: Eastern Cooperative Oncology Group performance status; EGJ: esophagogastric junction; PD-L1: programmed death ligand 1.

Supplementary Figure S1. GEP score distribution by histologic subtype (distribution scores based on the investigational in vitro diagnostic test)

Abbreviation: GEP, T-cell–inflamed gene expression profile.

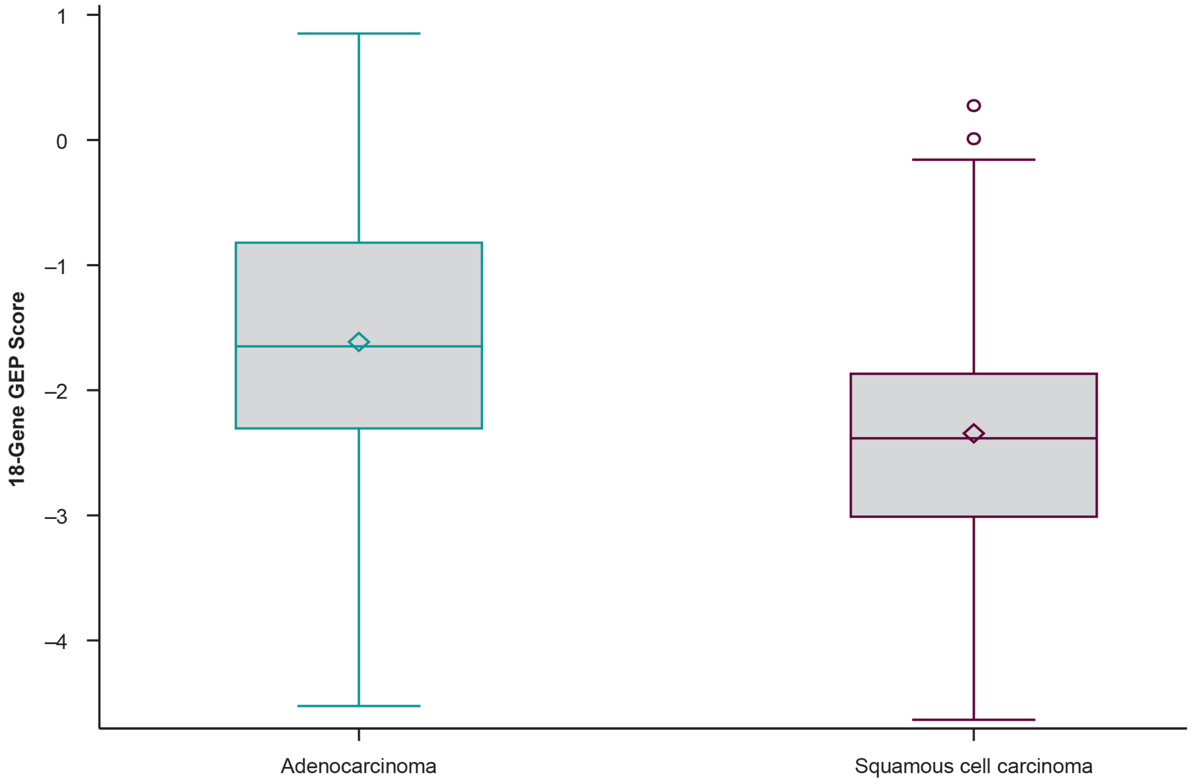

Supplementary Figure S2. PD-L1 expression by geographic region among patients with AC and SCC.

Abbreviations: AC: adenocarcinoma; CPS: combined positive score; PD-L1: programmed death ligand 1; SCC: squamous cell carcinoma.

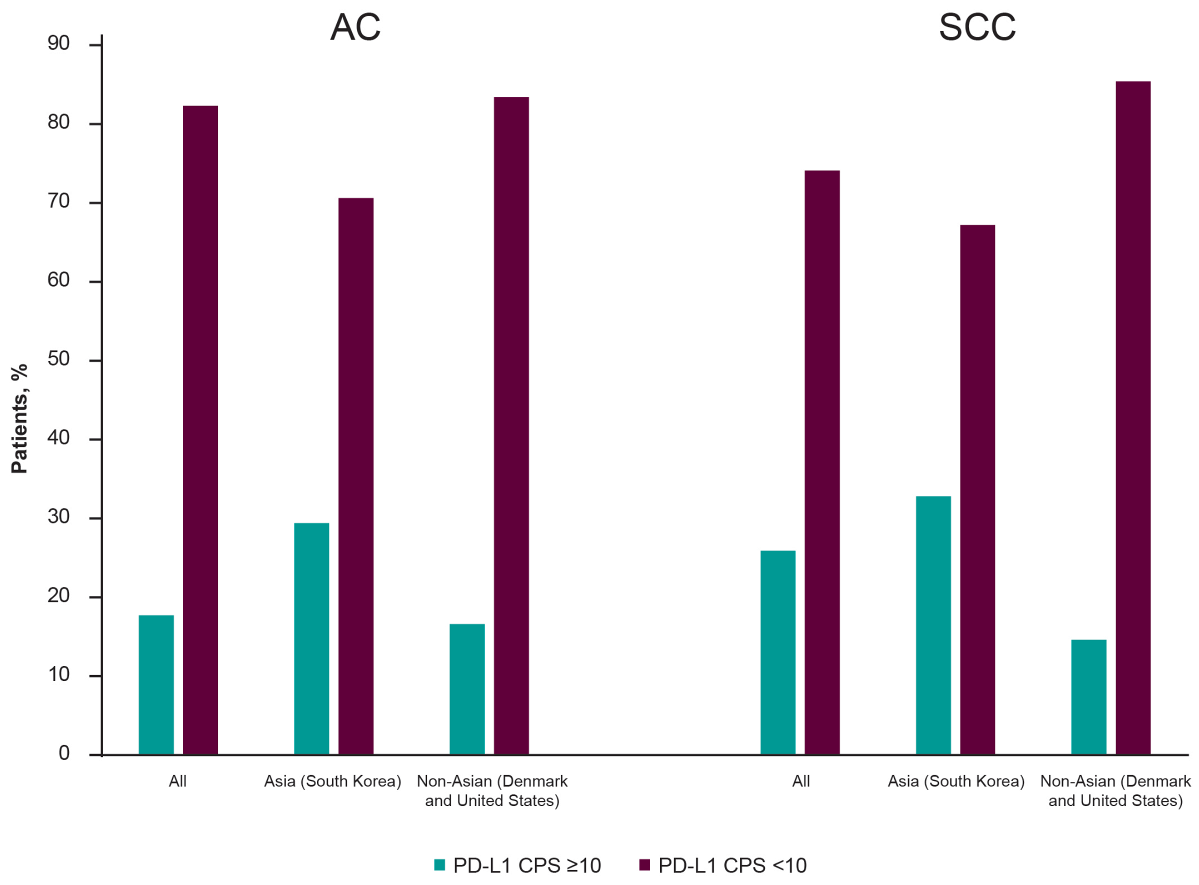

Supplementary Figure S3. Kaplan-Meier estimates of OS from the start date of first-line therapy by GEP status among (A) all patients, (B) patients with AC, and (C) patients with SCC.

Abbreviations: AC: adenocarcinoma; GEP: T-cell–inflamed gene expression profile; OS: overall survival; SCC: squamous cell carcinoma.

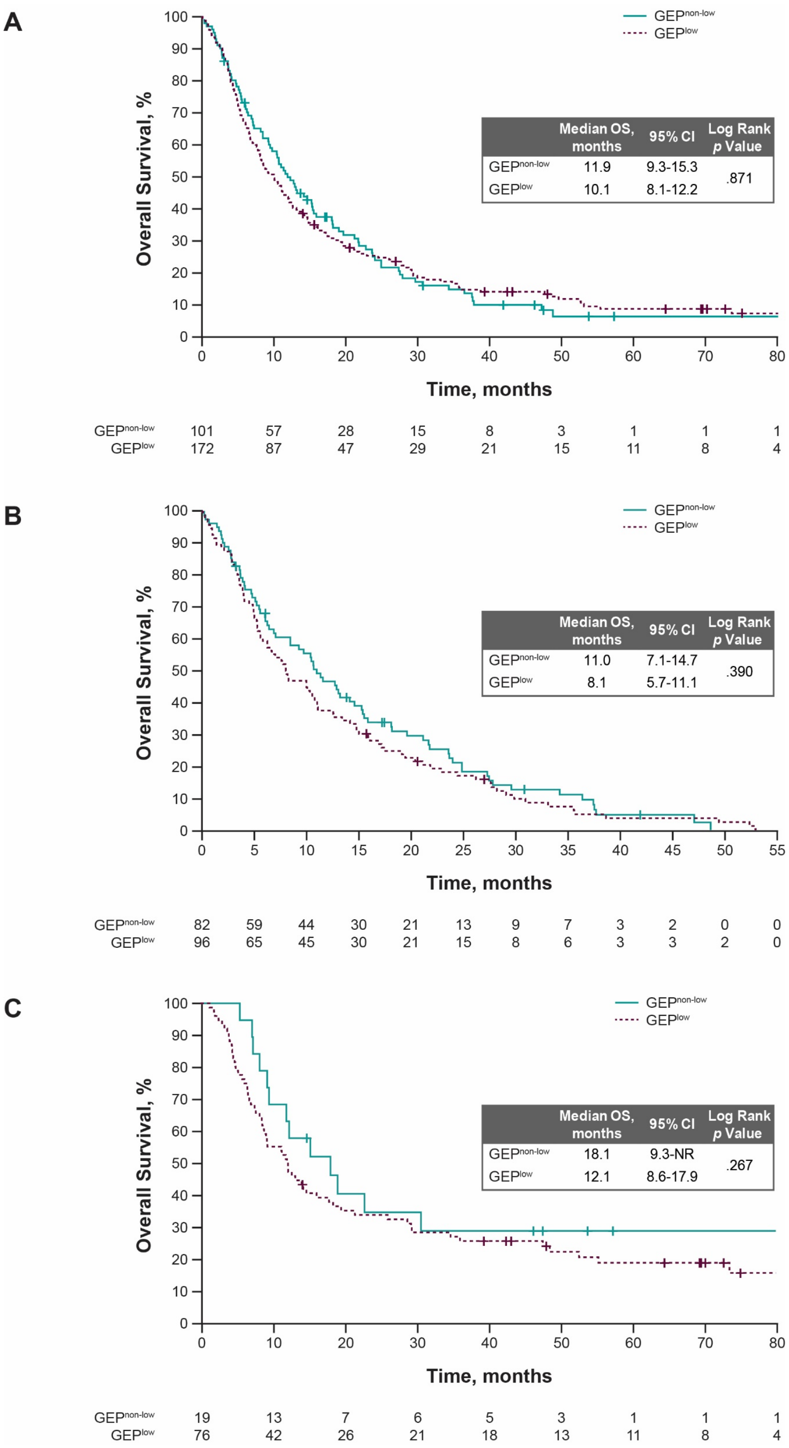
A
GEP non-low
GEP low
Overall Survival, %
Time, months
Median OS, months | 95% CI | Log Rank p Value
GEP non-low 11.9 9.3-15.3
GEP low 10.1 8.1-12.2
.871
GEP non-low 101 57 28 15 8 3 1 1 1
GEP low 172 87 47 29 21 15 11 8 4
B
GEP non-low
GEP low
Overall Survival, %
Time, months
Median OS, months | 95% CI | Log Rank p Value
GEP non-low 11.0 7.1-14.7
GEP low 8.1 5.7-11.1
.390
GEP non-low 82 59 44 30 21 13 9 7 3 2 0 0
GEP low 96 65 45 30 21 15 8 6 3 3 2 0
C
GEP non-low
GEP low
Overall Survival, %
Time, months
Median OS, months | 95% CI | Log Rank p Value
GEP non-low 18.1 9.3-NR
GEP low 12.1 8.6-17.9
.267
GEP non-low 19 13 7 6 5 3 1 1 1
GEP low 76 42 26 21 18 13 11 8 4

Supplementary Figure S4. Kaplan-Meier estimates of OS from date of diagnosis by GEP status among (A) all patients with stage I-III disease, (B) all patients with stage IV disease, (C) patients with stage I-III AC), (D) patients with stage IV AC, (E) patients with stage I-III SCC, and (F) patients with stage IV SCC.

Abbreviations: AC: adenocarcinoma; GEP: T-cell–inflamed gene expression profile; OS: overall survival; SCC: squamous cell carcinoma.

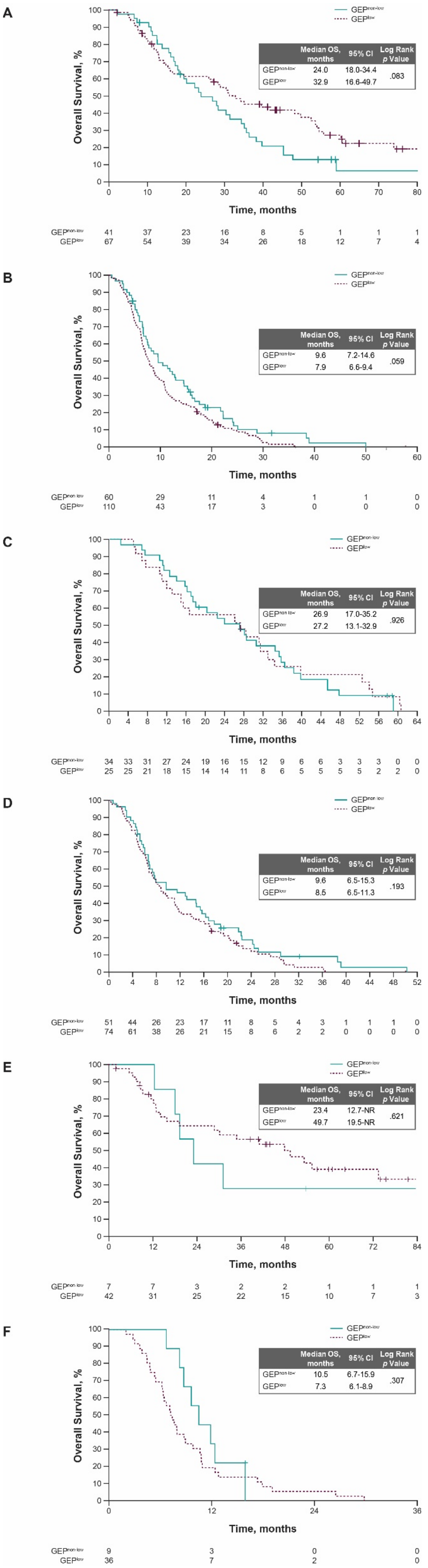
A
GEPnon-low
GEPlow
Median OS, months
95% CI
Log Rank p Value
GEPnon-low 24.0 18.0-34.4
GEPlow 32.9 16.6-49.7
.083
Overall Survival, %
Time, months
GEPnon-low 41 37 23 16 8 5 1 1 1
GEPlow 67 54 39 34 26 18 12 7 4
B
GEPnon-low
GEPlow
Median OS, months
95% CI
Log Rank p Value
GEPnon-low 9.6 7.2-14.6
GEPlow 7.9 6.6-9.4
.059
Overall Survival, %
Time, months
GEPnon-low 60 29 11 4 1 1 0
GEPlow 110 43 17 3 0 0 0
C
GEPnon-low
GEPlow
Median OS, months
95% CI
Log Rank p Value
GEPnon-low 26.9 17.0-35.2
GEPlow 27.2 13.1-32.9
.926
Overall Survival, %
Time, months
GEPnon-low 34 33 31 27 24 19 16 15 12 9 6 6 3 3 3 0 0
GEPlow 25 25 21 18 15 14 14 11 8 6 5 5 5 5 2 2 0
D
GEPnon-low
GEPlow
Median OS, months
95% CI
Log Rank p Value
GEPnon-low 9.6 6.5-15.3
GEPlow 8.5 6.5-11.3
.193
Overall Survival, %
Time, months
GEPnon-low 51 44 26 23 17 11 8 5 4 3 1 1 1 0
GEPlow 74 61 38 26 21 15 8 6 2 2 0 0 0 0
E
GEPnon-low
GEPlow
Median OS, months
95% CI
Log Rank p Value
GEPnon-low 23.4 12.7-NR
GEPlow 49.7 19.5-NR
.621
Overall Survival, %
Time, months
GEPnon-low 7 7 3 2 2 1 1 1
GEPlow 42 31 25 22 15 10 7 3
F
GEPnon-low
GEPlow
Median OS, months
95% CI
Log Rank p Value
GEPnon-low 10.5 6.7-15.9
GEPlow 7.3 6.1-8.9
.307
Overall Survival, %
Time, months
GEPnon-low 9 3 0 0
GEPlow 36 7 2 0

Supplementary Figure S5. Kaplan-Meier estimates for OS from date of diagnosis by PD-L1 expression (PD-L1 CPS ≥10 vs <10) and GEP status ($GEP^{non\text{-}low}$ vs GEP^{low}).

Abbreviations; CPS: combined positive score; GEP: T-cell–inflamed gene expression profile; OS: overall survival.

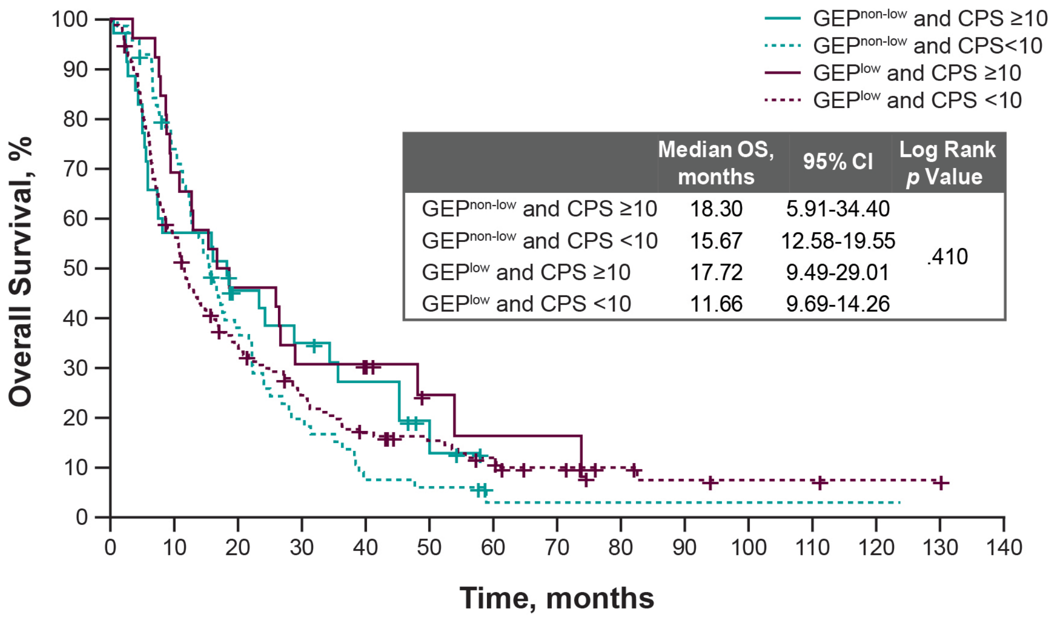

	Median OS, months	95% CI	Log Rank *p* Value
$GEP^{non\text{-}low}$ and CPS ≥10	18.30	5.91-34.40	.410
$GEP^{non\text{-}low}$ and CPS <10	15.67	12.58-19.55	
GEP^{low} and CPS ≥10	17.72	9.49-29.01	
GEP^{low} and CPS <10	11.66	9.69-14.26	

$GEP^{non\text{-}low}$ and CPS ≥10	35	20	13	10	7	3	0	0	0	0	0	0	0	0	0
$GEP^{non\text{-}low}$ and CPS <10	70	50	25	13	5	4	1	1	1	1	1	1	1	0	0
GEP^{low} and CPS ≥10	26	18	12	8	7	3	2	2	0	0	0	0	0	0	0
GEP^{low} and CPS <10	163	91	53	36	24	18	13	8	5	3	2	2	1	1	0
